# The multiple uses of artificial intelligence in exercise programs: a narrative review

**DOI:** 10.3389/fpubh.2025.1510801

**Published:** 2025-01-31

**Authors:** Alberto Canzone, Giacomo Belmonte, Antonino Patti, Domenico Savio Salvatore Vicari, Fabio Rapisarda, Valerio Giustino, Patrik Drid, Antonino Bianco

**Affiliations:** ^1^Sport and Exercise Sciences Research Unit, Department of Psychology, Educational Science and Human Movement, University of Palermo, Palermo, Italy; ^2^Department of Biomedical and Dental Sciences and Morphological and Functional Imaging, University of Messina, Messina, Italy; ^3^Department of Neurosciences, Biomedicine and Movement Sciences, University of Verona, Verona, Italy; ^4^Faculty of Sport and Physical Education, University of Novi Sad, Novi Sad, Serbia

**Keywords:** health, wellbeing, wellness, machine-learning, deep-learning, “artificial intelligence and movement”, sport, physical activity

## Abstract

**Background:**

Artificial intelligence is based on algorithms that enable machines to perform tasks and activities that generally require human intelligence, and its use offers innovative solutions in various fields. Machine learning, a subset of artificial intelligence, concentrates on empowering computers to learn and enhance from data autonomously; this narrative review seeks to elucidate the utilization of artificial intelligence in fostering physical activity, training, exercise, and health outcomes, addressing a significant gap in the comprehension of practical applications.

**Methods:**

Only Randomized Controlled Trials (RCTs) published in English were included. Inclusion criteria: all RCTs that use artificial intelligence to program, supervise, manage, or assist physical activity, training, exercise, or health programs. Only studies published from January 1, 2014, were considered. Exclusion criteria: all the studies that used robot-assisted, robot-supported, or robotic training were excluded.

**Results:**

A total of 1772 studies were identified. After the first stage, where the duplicates were removed, 1,004 articles were screened by title and abstract. A total of 24 studies were identified, and finally, after a full-text review, 15 studies were identified as meeting all eligibility criteria for inclusion. The findings suggest that artificial intelligence holds promise in promoting physical activity across diverse populations, including children, adolescents, adults, older adult, and individuals with disabilities.

**Conclusion:**

Our research found that artificial intelligence, machine learning and deep learning techniques were used: (a) as part of applications to generate automatic messages and be able to communicate with users; (b) as a predictive approach and for gesture and posture recognition; (c) as a control system; (d) as data collector; and (e) as a guided trainer.

## Introduction

Physical activity has been recognized as an effective approach for enhancing public health. Promoted by multiple medical organizations, it is helpful in the prevention and treatment of various diseases (1). It is essential for improving individuals’ overall health by providing significant advantages, including the reduction of chronic illness risk and the enhancement of mental well-being (1–4). The potential of artificial intelligence in training, exercise, physical activity, and health programs stems from its capacity to evaluate, compute, and reveal findings. Its use in medicine is increasingly broadening, presently manifesting in social media, video games, smartphones, and smartwatches (5–9). This can make us realize how easy it is to find ourselves in the context of the presence of artificial intelligence, which can push us to use it to our advantage. Therefore, in the context of training or health programs, it becomes easy to think about the use of artificial intelligence as a way to communicate with users via messaging apps, as a potential predictive and recognition tool, or as a device for data analysis and collection (10–12).

Artificial intelligence is based on algorithms that enable machines to perform tasks and activities that generally require human intelligence, and its use offers innovative solutions in various fields (13, 14). The use of artificial intelligence can be important in public health. In a systematic review of the use of chatbots (artificial intelligence systems) for healthcare applications, Xu et al. (15) describe integrating these elements into clinical practice, equipping healthcare workers with a valuable resource while preserving the fundamental function of human involvement in medical care (15). Artificial intelligence includes machine learning, a subset that uses statistical techniques and computational algorithms to analyze data and discern patterns (16–18). Machine learning can be classified by task type into supervised learning, unsupervised learning, and reinforcement learning (16, 19).

Supervised learning emphasizes predictive tasks. It utilizes labeled samples to train algorithms that identify or forecast specified outcomes. It is very effective for formulating risk and prognosis scores to identify individuals who could benefit from preventive or tailored therapies (20–23). Banker et al. utilized supervised learning with adaptive, multi-step algorithms to analyze wearable accelerometer data and assess the influence of physical exercise on biological aging (24).

Unlike supervised learning, unsupervised machine learning was developed to identify novel patterns and correlations in irregularly sampled data without the utilization of human-generated labels.

Unsupervised learning does not focus on a particular identification task but instead tries to uncover the overall structure underlying a dataset, discovering possible trends, correlations, and associations along both spatial and spectral domains. This method offers an exploratory data analysis without focusing on specific areas of interest. The structure that is found in the data can be used to assist human interpretation, but it can also help in reducing the computational load for subsequent analysis (25–28). Unsupervised learning could be used to identify patterns in physical activity data without predefined labels. Gupta et al. used unsupervised learning to identify patterns of COVID-19 symptoms associated with long and short COVID-19 in a nonhospitalized cohort, to assess the presence of distinct patterns of physical activity trajectory, and to evaluate an association between COVID-19 and patterns of physical activity trajectory (29).

Reinforcement learning is a machine learning methodology in which an agent optimizes actions via trial and error, gaining incentives for favorable behaviors within an interactive environment. In contrast to supervised learning, it depends on a progressive reward system instead of direct instructions (30, 31). Reinforcement learning could be used as a way to communicate with users via smartphones. Yom-Tov et al. developed a mobile application that operates in the background on the smartphones of diabetes patients, monitoring their physical activity levels (32). They used a reinforcement learning algorithm that assessed which SMS message would likely increase the participants’ physical activity the next day, and subsequently, that message was sent to them (32).

Deep learning, a kind of machine learning, is the predominant technique in numerous applications. It employs multilayer neural networks to autonomously acquire data representations, converting input into various levels of abstraction. This technique is good for handling large-scale, high-density data (13, 33, 34). In a recent study, Hamid et al. used deep learning models to accurately approximate energy cost, in terms of metabolic equivalents of physical activity, using sensor readings from wearable accelerometers in children (35). The authors analyzed activities encompassing prevalent locomotor and object control motions in children. Their analysis examined the influence of sensor placement on the model’s predictive efficacy, providing recommendations for optimal sensor sites for each activity category (35).

Therefore, the use of artificial intelligence in training, exercise, physical activity and health programs can vary, depending on the type of artificial intelligence used. Given the rapid integration of artificial intelligence in several health domains, this narrative review aims to understand how artificial intelligence is being used to promote physical activity, training, exercise, and health outcomes, filling a critical gap in understanding the practical applications.

## Materials and methods

### Search strategy

This narrative review was carried out following the narrative checklist (36). The PubMed online database was used as a research tool, on August 16, 2024, using the following strings: “Artificial Intelligence and physical activity,” “Artificial Intelligence and fitness,” “Artificial Intelligence and movement,” “Artificial Intelligence and training,” “Machine learning and physical activity,” “Machine learning and fitness,” “Machine learning and movement,” and “Machine learning and training.” The RCTs option has been marked for the search performed on PubMed.

### Study selection

Only RCTs written in English were included. Inclusion criteria: all Randomized Controlled Trials (RCTs) that use artificial intelligence to program, supervise, manage, or assist physical activity, training, exercise, or health programs. Only studies published from January 1, 2014, were considered. Exclusion criteria: all the studies that used robot-assisted, robot-supported, or robotic training were excluded. After extraction, all articles reviewed from the PubMed online database were entered into EndNote 21 software. In the first stage, two investigators worked independently by removing duplicates and analyzing articles by title and abstract. In the second phase, all included articles were reviewed through a full-text reading to assess whether they fell within the inclusion criteria. The opinion of a third researcher was considered in case of disagreement between the two researchers.

### Data extraction

Data on “date and author, participants, artificial intelligence function, intervention, and outcomes” were collected, put into a Microsoft Word spreadsheet, and then analyzed in the discussion.

## Results

### Studies’ identification

**Figure 1:**
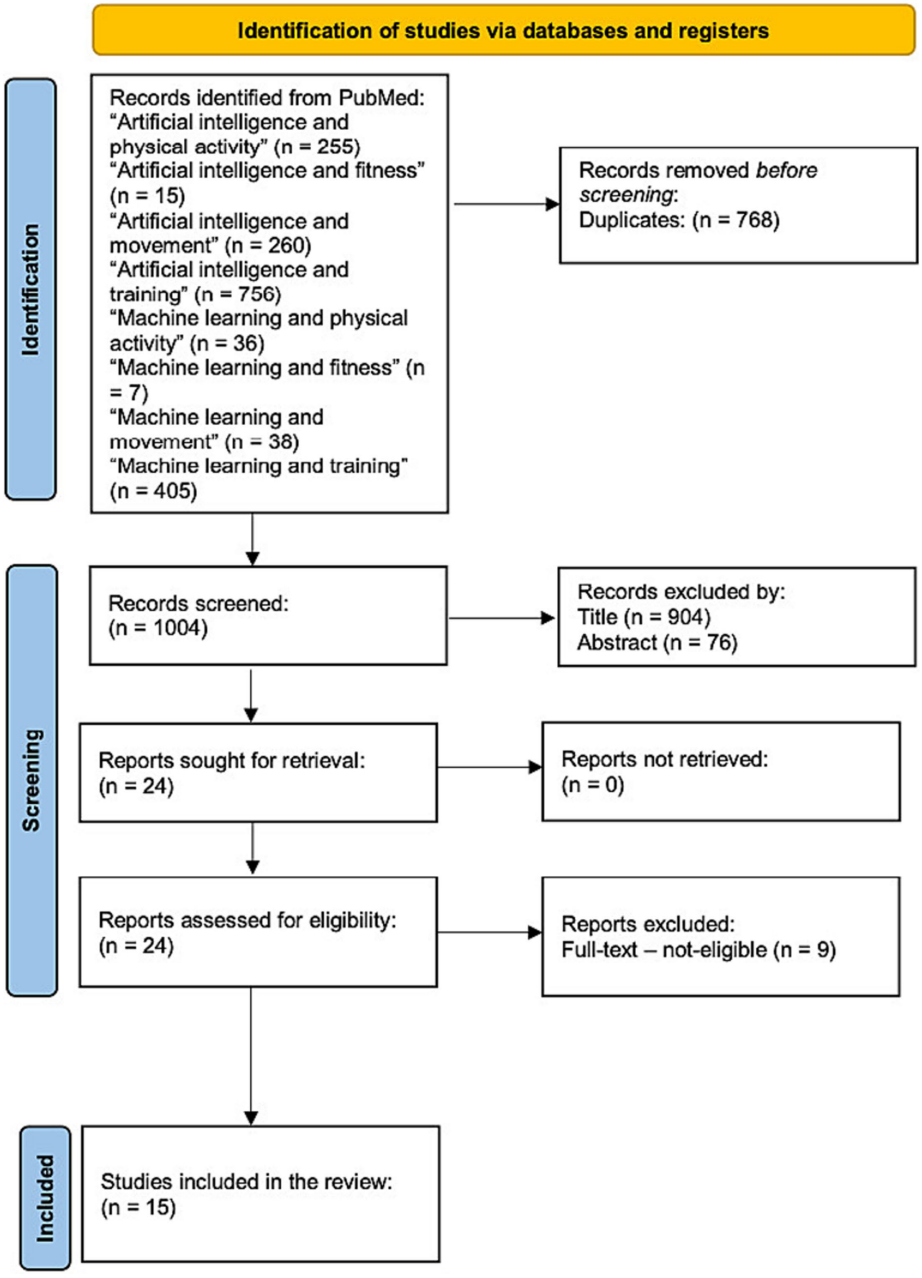
Study selection and eligibility screening flow diagram.

A number of 1772 studies were identified. After an initial phase, where duplicates were cleared, a screening was then conducted on the remaining 1,004 articles based on title and abstract. A total of 24 studies were identified, and finally, after a full-text review, 15 studies were identified as meeting all eligibility criteria for inclusion. Figure 1 provides a detailed flow diagram outlining the process of the study identification, screening, and inclusion (37). Table 1 summarizes the characteristics of the included studies, including participants, artificial intelligence functionalities, intervention, and outcomes.

**Table 1:** The list of studies included in the narrative review.

Date and author	Participants	AI Function	Intervention	Outcomes
2021, Anan (38)	Middle Aged Women and Men Intervention group *n* = 48 Control group *n* = 46	Texting messages and notifications	Stretching, maintaining good posture, mindfulness	Subjective assessment of the degree of pain, subjective rate of pain improvement
2023, Bates (52)	Adults Control *n* = 9 Train *n* = 13 Clin *n* = 27 Combo *n* = 20	Artificial intelligence guided training	Moderate-intensity resistance training	6-min walking test (6MWT), Biering-Sorenson’s exam, Numeric Pain Rating scale (NPRS), Tampa scale of kinesiophobia (TSK), Patient Reported Outcomes Measurement Information System (PROMIS) physical function, and PROMIS pain interference
2023, Bizhanova (39)	Adults with overweight or obesity Self-monitoring of diet, physical activity, and weight (SM) *n* = 251 Self- monitoring of diet, physical activity, and weight combined with daily tailored feedback messages (SM + FB) *n* = 251	Text generation and machine learning prediction models for the average percentage of adherence to the physical activity goal	Self-monitoring of diet, physical activity, weight and daily tailored feedback messages	Fitbit tracker (MVPA), Self-efficacy and Exercise Habits Survey, Center for Epidemiologic Studies Depression (CES-D), and Tanita scale
2023, Cao (40)	Students (age range of 8–13) Intervention group *n* = 60 Control group *n* = 56	Machine learning-based approach for postural recognition – back-propagation neural network (BPNN) in the context of deep learning to analyze soccer player’s actions	Functional strength training	10- and 30-meter sprint tests (speed), Illinois agility test and 5*25-meter shuttle run (sensitivity), throws and set kicking drills (strength), analysis of kicking actions (BPNN)
2024, He (47)	Older adult Self-determined sequence exercise program group (SDSG) *n* = 34 Strength training group (STG) *n* = 30 Control group *n* = 30	Machine learning classification models - explainable artificial intelligence (XAI) – and a stacking model to predict whether sarcopenia could regress in subjects after the intervention	Strength training and Yi Jin Jing exercise	Hydraulic hand dynamometer, 6-meter gait speed, appendicular skeletal muscle mass (ASM) – L3 skeletal muscle area (L3SMA), L3 skeletal muscle density (L3SMD), L3 skeletal muscle interstitial fat area (L3SMFA), L3 skeletal muscle interstitial fat density (L3SMFD), relative skeletal muscle mass index (RSMI), muscle fat infiltration (MFI)
2021, Kristoffersen (41)	Adults with unilateral upper limb absence: Game group *n* = 2 Conventional group *n* = 2	Control system composed by feature extraction, regression between EMG features and hand movement commands, post-processing of movement commands to improve usability and suppress possible errors and a feed-forward neural network was used as a regressor to compute hand movement commands from the pre- processed EMG data	Gaming training and conventional training using a computer program	Southampton Hand Assessment Procedure (SHAP), The Clothespin Relocation Test (CRT)
2021, Lao (48)	Older adult Exercising wearing the bracelet *n* = 20 Exercising without the bracelet *n* = 20 Control *n* = 20	Data collect by artificial intelligence sports bracelets	Cardiopulmonary endurance training, muscle strength training, flexibility training, and balance training	Exercise motivation scale (EMS), and Borg rating of perceived exertion scale (RPE scale)
2023, Marcuzzi (49)	Adults Usual care group *n* = 97 App group (selfBACK) *n* = 99 E-Help group *n* = 98	Texting messages and notifications	Video instructions for strength and flexibility exercises, daily educational content, and recommendations of physical activity	Musculoskeletal Health Questionnaire (MSK-HQ), Roland-Morris Disability Questionnaire (RMDQ), Numeric Rating Scale (NRS), Pain Self-Efficacy Questionnaire (PSEQ), Brief Illness Perception Questionnaire (BIPQ), EuroQol 5-dimension questionnaire and Global Perceived Effect scale
2017, Mendoza (42)	Fifth grade students Intervention *n* = 24 Control *n* = 30	Machine learning algorithms to identify cycling behaviors	Bicycle train program	Questionnaire to assess the percentage of trips made to school by cycling and MVPA (accelerometer and GPS)
2022, Nakata (43)	Adults Intervention *n* = 71 Control *n* = 69	Texting messages and notifications	Advice based on the diet, exercise, sleep, mood, and weight recorded by users	Weight scale, test kit (blood), brief self-administered diet history questionnaire (BDHQ), triaxial accelerometer (for the intensity of physical activity based on metabolic equivalents from published algorithm)
2023, Nordstoga (44)	Adults ≤12 weeks Control *n* = 93 selfBACK *n* = 101 ≥12 weeks Control *n* = 136 selfBACK *n* = 131 Low (≤5) Control *n* = 131 selfBACK *n* = 145 Low (≥5) Control *n* = 98 selfBACK *n* = 87	Texting messages and notifications	Video instructions for strength and flexibility exercises, daily educational content, and recommendations of physical activity	Roland-Morris Disability Questionnaire (RMDQ), Numeric Rating Scale (NRS), Pain Self-Efficacy Questionnaire, and Global Perceived Effect scale
2022, Oh (46)	Children and adolescents with obesity: SUKIA games group *n* = 12 NINS group *n* = 12	Convolutional neural network (CNN) in a deep-learning algorithm for gesture recognition	Boxing, lunging, squatting, side-bending, upper extremity stretching, arm and jumping exercise (SUKIA game) and Nintendo Switch (NINS) health-oriented role-playing game	Calorie consumption, VO2max, 6-min walking test (6MWT), body mass index (BMI), and the Borg rating of perceived exertion scale (RPE), post-questionnaire questions on perceived exercise effectiveness, motivation and fun
2022, Øverås (45)	Adults Multimorbidity NO Usual care group *n* = 68 selfBACK group *n* = 81 Multimorbidity YES Usual care group *n* = 161 selfBACK group *n* = 151 No. of co.occurring musculoskeletal pain sites 0–1 Usual care group *n* = 89 selfBACK group *n* = 101 No. of co.occurring musculoskeletal pain sites 2+ Usual care group *n* = 140 selfBACK group *n* = 131	Texting messages and notifications	Video instructions for strength and flexibility exercises, daily educational content, and recommendations of physical activity	Roland-Morris Disability Questionnaire (RMDQ), Health-related quality of life (EQ-5D), Perceived Stress Scale, Patient Health Questionnaire-8, General Health, Brief Illness Perception Questionnaire (BIPQ), Pain Self-Efficacy Questionnaire (PSEQ), Saltin-Grimby Physical Activity Level Scale, and Patient’s Global Perceived Effect (GPE)
2022, Wei (50)	Older adults Yi Jin Jing and resistance training (YR) group *n* = 30 Resistance training (RT) group *n* = 30 Control group *n* = 30	Machine learning classification models - explainable artificial intelligence (XAI) - and a stacking model to predict whether sarcopenia could regress in subjects after the intervention	Yi Jin Jing exercise and resistance training	Hydraulic hand dynamometer, 6-meter gait speed, and appendicular skeletal muscle mass (ASM) – L3 skeletal muscle area (L3SMA), L3 skeletal muscle density (L3SMD), L3 skeletal muscle interstitial fat area (L3SMFA), L3 skeletal muscle interstitial fat density (L3SMFD), relative skeletal muscle mass index (RSMI), muscle fat infiltration (MFI)
2023, Wei (51)	Older adults Wu Qin Xi group (WQX) *n* = 52 Strength and endurance training group (SE) *n* = 57 WQXSE *n* = 54	Explainable artificial intelligence (XAI) to predict whether subjects could reverse frailty	Wu Qin Xi exercise, strength and endurance training	Unintentional weight loss, self-reported fatigue, physical activity scale for older adult in Chine (PASE-C), Timed up-and-go test (TUGT), hydraulic hand dynamometer, 6 min walk test (6MWT), 10 m maximum walking speed (10 m MWS), and 10 m walking time (gait velocity)

### Study characteristics

A total of 2,626 subjects were evaluated, in total, in nine studies (38–46) participants were divided into two groups, in five studies (47–51) into three groups, and in one study (52) into four groups. In addition, artificial intelligence was used to send messages and notifications in six studies (38, 39, 43–45, 49), as a predictive model in four studies (39, 47, 50, 51), as a model to analyze gestures and postures in three studies (40, 42, 46), as a data collector in one study (48), and as a training and control guide in two studies (41, 52). Regarding the interventions, stretching exercises, maintaining good posture and mindfulness (38) were provided; notifications regarding self-monitoring diet, physical activity, weight and personalized daily feedback messages were suggested in the included studies (39); also stretching (46), lunging (46), boxing (46), side-bending (46), squatting (46), jumping exercise (46) were offered; continuing with resistance (50, 52), strength (40, 44, 45, 47–49, 51), gaming (41, 46), cardiopulmonary endurance (48), endurance (51), flexibility (44, 45, 48, 49), balance (48) training programs, and a bicycle train program (42) were conducted; Yi Jin Jing (47, 50) and Wu Qin Xi (51) exercises were performed; daily education content and recommendations of physical activity (44, 45, 49), and advice on diet (43), exercise (43), sleep (43), mood (43), and weight (43) were also suggested.

## Discussion

This narrative review reveals that the use and application of artificial intelligence in physical activity, exercise, training contexts is still emerging, particularly when compared to its extensive use in medical fields (53–57). The rapid advancements in artificial intelligence, particularly from 2017 to 2024 as reflected in the included studies, underscore its evolving role in wellness and health intervention. The included studies utilized diverse artificial intelligence functionalities, ranging from artificial intelligence messaging systems to predictive models and data collection, and from artificial intelligence systems for gesture recognition, analysis, and control to artificial intelligence-guided training methods. These approaches targeted outcomes such as pain reduction, predictive events, and performance improvements, demonstrating the versatility of artificial intelligence to promote physical activity and health programs.

### Artificial intelligence function

#### Texting messages and notifications

In their study, Anan et al. (38) looked at improving pain/stiffness symptoms in the neck/shoulder districts in workers and used an interactive artificial intelligence-assisted health promotion system via a messaging app to communicate with them. Anan et al. (38) programmed the artificial intelligence to send messages to workers both with exercise instructions and to suggest what they could do to improve symptoms. While Nakata et al. (43) developed a smartphone healthcare application where users could record physical activity, daily diet, sleep quality, and mood to calculate their dietary intake and give recommendations using artificial intelligence technology. Instead, Nordstoga et al. (44), Marcuzzi et al. (49), and Øverås et al. (45) used the selfBACK app in their studies. The app contained three major components of self-management such as video instructions for flexibility and strength exercises, recommendations of physical activity, and daily educational content. Weekly recommendations for self-management were delivered for each of the components and were tailored to individual characteristics, progression, and symptoms using case-based reasoning methodology, a branch of knowledge-driven artificial intelligence. Also, the app included tools such as mindfulness audios, setting goals, sleep reminders, and low back pain (LBP) relief exercises. Subjects were given encouraging push notifications, activated based on their self-management behavior, to motivate and reinforce their desired behavior. Likewise, Bizhanova et al. (39) used the SMARTER app to communicate with users by sending feedback messages. The application allowed subjects to be sent up to three daily feedback messages tailored to the self-monitoring data collected. The algorithm randomly sent messages to the participants’ smartphones during the morning, afternoon, and evening hours.

#### Predictive model

Wei et al. (50, 51) and He et al. (47) in three different studies, used an explainable artificial intelligence (XAI) in order to predict if subjects could reverse frailty, 8 and 10 classical machine learning classification models, and a stacking model in order to predict if sarcopenia might regress in subjects after the intervention. Also, Bizhanova et al. (39) used machine learning prediction models for the average percentage of adherence to physical activity (PA) goal.

#### Device to analyze gestures and postures

Cao et al. (40) investigated the effect of functional strength training on football players’ abilities and used machine learning to examine players’ movements. Specifically, Cao et al. (40) used machine learning technics, in particular, back-propagation neural network (BPNN) in the context of deep learning, to investigate the actions of soccer players. They used sensitivity, force, and movement speed as BPNN input vectors to confront images of players’ movements, while they used the standard movements and similarity among soccer actions as output results to improve training efficiency. Meanwhile, Oh et al. (46) used an artificial intelligence-program gaming technique, they were concerned with studying the effects of total body motion movement through Super Kids Adventure (SUKIA), a gesture recognition app based on artificial intelligence using a convolutional neural network (CNN) in deep learning algorithm, and the Nintendo Switch (NINS) in adolescents with obesity. Instead, Mendoza et al. (42) used machine learning algorithms to detect cycling patterns of behavior. They developed and validated machine learning algorithms based on simultaneously recorded accelerometer and GPS data to identify the timing and activity of cycling in children.

#### Data collector

Lao et al. (48) used artificial intelligence smart bracelets to collect data and track physiological records during exercise so that users could obtain a clearer idea of their physical status during exercise.

#### Training and control guide

Like Oh et al. (46), Kristoffersen et al. (41) used an artificial intelligence program game technique. Specifically, Kristoffersen et al. (41) were concerned with comparing game training with conventional training for subjects with upper limb absence (ULA) using a machine learning-based control system that consisted of regression between electromyogram (EMG) features and hand movement commands, post-processing of movement commands to improve usability and suppress possible errors, and feature extraction. While Bates et al. (52) used an artificial intelligence-guided training. Precisely, the groups of intervention were conducted on a Tonal exercise trainer. Bates et al. (52) determined resistance selection with the artificial intelligence calibration by the Tonal trainer for all individuals. During the calibration, all subjects executed three repetitions of side pulls, deadlifts, overhead press, and bench press at maximum force, and depending on the quantity of power delivered in these tasks, the Tonal artificial intelligence trainer estimated and suggested resistances for all tasks in the training program. Based on data collected in real-time, this software algorithm provides real-time feedback to an active user. Bates et al. (52) used this algorithm to monitor variables such as speed, range of motion, and power of performance to make automatic adjustments to the amount of unique resistance for all subjects according to their performance. In addition, the artificial intelligence-guided tasks were supervised by the research team’s physical trainers to provide safety for the participants and give feedback on the subjects’ form and technique (52).

### Exercise programs

#### Strength, flexibility, and endurance training

A total of seven studies used strength, flexibility, and endurance exercises (40, 44, 45, 47–49, 51). Specifically, educational content and physical activity recommendations were also suggested in three articles (44, 45, 49), balance exercises were also performed in one article (48), and finally, Yi Jin Jing and Wu Qin Xi exercises were also performed in two different studies (47, 51).

In the study by Cao et al. (40), the experimental group that performed functional strength training showed improvements in some performance compared to the baseline regarding speed, sensitivity, and strength performance. However, Cao et al. (40) observed no statistically meaningful differences in performance between the control group and the experimental group, especially in speed performance and set kicking test. As previously mentioned then no meaningful differences between the two groups were observed in either the 10-meter or 30-meter sprint test but Cao et al. (40) found significant between-group differences regarding the Illinois running test, the 5*25-meter shuttle running test, and the throwing test. Finally, no major between-group differences were noted in kicking actions, measured with backpropagation neural network (BPNN), at baseline and after the experimental study, in fact, at the baseline, the accuracy of the experimental group’s kick actions was 73.2%, while that of the control group was 74.3%, whereas at post-test, the experimental group achieved an 83.4% accuracy, while the control group reached 84.1% (40). The lack of meaningful differences between groups in some performance metrics suggests the need for further exploration of artificial intelligence’s role in functional strength training (40). In the studies by Nordstoga et al. (44), Øverås et al. (45), and Marcuzzi et al. (49) video instructions for strength and flexibility exercises were offered. Specifically, Nordstoga et al. (44) showed that 185 subjects initially exhibited LBP intensity exceeding five on the Numeric Rating Scale (NRS) scale, whereas 267 experienced LBP episodes lasting longer than 12 weeks. After three months, the efficacy of the program was comparable irrespective of pain length; however, selfBACK users reported a 0.2-point decrease in LBP-related disability for low-intensity pain (≤5 NRS) and a 1.8-point decrease for high-intensity pain (≥5 NRS). Meanwhile, Øverås et al. (45) observed that persons with multimorbidity or numerous musculoskeletal pain sites were generally older, predominantly female, and had greater pain intensity, diminished physical activity, and decreased work levels. Prevalent long-term ailments encompassed mental health disorders and gastrointestinal problems, with hips and thighs frequently identified as co-occurring locations of musculoskeletal discomfort. The average number of co-occurring musculoskeletal pain locations was 2.14 in the intervention group and 2.34 in the control group. Moreover, Øverås et al. (45) did not find, in any of the outcomes studied, evidence regarding whether multimorbidity could modify the effect of the intervention. Although, the adjusted mean difference in the Roland-Morris Disability Questionnaire (RMDQ) score comparing the two groups at 3 months showed a slight positive effect for subjects with multimorbidity at baseline compared with those without multimorbidity, whereas the effect ended at 9 months of follow-up. About RMDQ, they noted that subjects with ≥2 LCTs had less reduction than subjects without any or one LTC plus LBP. Øverås et al. (45) also observed that participants with MSK co-occurring pain sites, LBP, and ≥ 4 pain sites, showed less improvement than participants with less pain co-occurring sites. Øverås et al. (45) observed that all groups showed minimum enhancement for every outcome (secondary outcomes) across all time points. Specifically, subjects without LTC and with LBP with 0–1 additional MSK pain site showed greater improvement in measures of general health, stress, and depression, while subjects with LBP and two or more LTC and four or more additional MSK pain sites showed minor enhancement in perceived self-efficacy, perceived global affect, and perceived illness. Finally, concerning the EuroQol 5-dimension questionnaire (EQ5D), Øverås et al. (45) found similar small increases across all groups over time. While, Marcuzzi et al. (49) observed that the app group exhibited superior Musculoskeletal Health Questionnaire (MSK-HQ) ratings at 3 months relative to the usual care and e-Help groups, although the differences were minimal by 6 months. At 3 months, 59.0% of app users indicated a ≥ 4-point enhancement in MSK-HQ, in contrast to 44.2% for usual care and 46.8% for e-Help (49). Secondary outcomes revealed no significant differences across groups, with the exception of GPE scores, which were elevated in the app group at 3 months (49). These studies point out that apps have the ability to send feedback and advice to a large number of users, but the fact that users have to perform specific exercises on their own, despite the feedback received through the app, could be a limitation (44, 45, 49). Instead, in the study by He et al. (47) strength training and Yi Jin Jing exercises were performed. They observed highly significant group-time interactions in handgrip strength, L3 skeletal muscle density (L3SMD), RSMI, and L3SMA. Specifically, at week 24, grip strength, L3SMA, RSMI, and L3SMD improved significantly in the self-determined sequence group (SDSG) and strength training group (STG). In addition, the SDSG group reached meaningfully higher RSMI and handgrip strength compared with the control group and STG group at week 24 (47). However, no significant interactions were found in L3 skeletal muscle interstitial fat area (L3SMFA), L3 skeletal muscle interstitial fat density (L3SMFD), and Muscle Fat Infiltration (MFI). About the stacking model, He et al. (47) showed that it had a high accuracy and that the handgrip strength was a major contributor to the model’s prediction performance. Finally, Wei et al. (51) found no statistically meaningful differences in subjects’ pre-intervention levels of fitness in each group, but after the intervention, they observed meaningful interaction time-group effects in the 10 m Maximum Walking Speed (10 m MWS) and grip strength. Also, Wei et al. (51), after 24 weeks of Wu Qin Xi exercises, and strength and endurance training, found a significant improvement in grip strength between the individuals in the group that performed both Wu Qin Xi and strength training (WQXSE) and those that performed strength training (SE) compared with subjects in the Wu Qin Xi group (WQX), and the WQXSE group also showed meaningful enhancement in Timed Up and Go Test (TUGT) when compared with the WQX group. In addition, they noted that the SE group had a meaningful enhancement in the 6-min walk test (6MWT), but the WQXSE group had meaningful enhancement in the 10 m MWS when compared to both WQX and SE groups (51). Regarding the results of the machine learning models, Wei et al. (51) found that the model’s multiple assessment metrics showed that the stacking model performed positively to predict successfully subjects’ frailty status after the intervention. Wei et al. (51) noted through this model, how handgrip strength exhibited the greatest contribuition between every characteristic, followed by 10 m WMS. This might suggest that when targeting to improve the health of the physically frail older adult, special focus on improving the performance of handgrip strength and 10 m MWS should be given. These studies suggest that artificial intelligence, after analyzing a set of data, can be used as an excellent predictive model (47, 51). Finally, Lao et al. (48) noted that before the experiment, there were no meaningful differences between the three groups. However, after 12 weeks of muscle strength training, cardiopulmonary endurance, balance, and flexibility training, the group that trained using the sports bracelets and the group that exercised without the sports bracelets showed significant improvements on the exercise motivation scale. These improvements were slightly greater in the group that trained using the bracelet than in the group that trained without the bracelet. The control group showed no significant changes. Artificial intelligence sports bracelets have proven to be excellent devices for collecting data on distance traveled, time spent exercising and sleeping, and calculating calories consumed based on the amount of exercise (48).

#### Resistance training

A total of two studies performed resistance training, of which Yi Jin Jing exercises were performed in one study.

In another study, Wei et al. (50) showed how, after 24 weeks of Yi Jin Jing exercise and resistance training, 27.8% of subjects had a reversal of sarcopenia, specifically 52.0% of participants in the group that performed both Yi Jin Jing exercise and resistance training (YR) (13/30) and 48.0% of subjects in the group that performed resistance training (RT) (12/30). They also noted that subjects in YR and RT had meaningful enhancements in L3 skeletal muscle area (L3SMA), Relative Skeletal Muscle Mass Index (RSMI), and handgrip strength, and in particular, the YR group when compared to the RT and control group, showed significantly better RSMI and L3SMA. Finally, the stacking model was able, with 85.7% accuracy, to predict sarcopenia in the older adult (50). Also in this study, as in the previous one, we see that artificial intelligence can be used as an excellent predictive model (50). Instead, Bates et al. (52), using artificial-intelligence-guided moderate-resistance training on subjects suffering from LBP, noted that for the Biering-Sorenson Examination, the overall time duration of isometric extensor resistance enhanced from baseline to week 8 in the group with subjects actively seeking clinical care (CLIN) and in the group with subjects actively seeking clinical care and artificial intelligence-supervised-guided training (COMBO). In addition, for the 6MWT, Bates et al. (52) observed that the distance walked had increased at week 8 for the CLIN group, had neared significance for the COMBO group, and had not changed either for the group with subjects who were not clinical and who and who were not given intervention (CONTROL) or for the group with subjects who had artificial intelligence-supervised-guided training (TRAIN). Continuing, Bates et al. (52) noted that the Numeric Pain Rating Scale (NPRS) score was decreased in the TRAIN group and seemed to tend to decline in the CLIN and COMBO groups. Regarding the Tampa Scale of Kinesiophobia (TSK) score, it was observed that the CLIN group had decreased scores at week 8. Regarding Patient Reported Outcomes Measurement Information System (PROMIS) physical function scores, Bates et al. (52) observed an increase at week 8 in the CONTROL, TRAIN, and COMBO groups, while the CLIN group had no change. Finally, from PROMIS pain interference scores, Bates et al. (52) noted a decrease from baseline to week 8 in the TRAIN, CLIN, and COMBO groups, while no meaningful PROMIS scores of pain interference were found in the CONTROL group. The study supports the possible role of artificial intelligence-guided resistance training in alleviating LBP and improving function and pain, highlighting possible gains and utility for LBP prevention if combined with clinical care (52).

#### Stretching and game training

In a total of three studies, stretching, and game training exercises were performed, specifically in one study, boxing, squatting, side-bending, lunging, arm, and jumping exercises were also performed (38, 41, 46).

In the study by Anan et al. (38), participants’ adherence was 92%. At the end of the health program, in which stretching exercises were also suggested, Anan et al. (38) found an improvement in neck-shoulder stiffness-pain or LBP in the experimental group compared with the control group, furthermore, the percentage of subjects who had symptoms of severe importance decreased for 77 to 33% in the experimental group, while from 76 to 67% in the control. Finally, after 12 weeks of the artificial intelligence-assisted health program, 75% of the experimental group perceived improvements versus only 7% in the control group (38). This highlights the potential of artificial intelligence-based messaging systems to enhance the adherence and achieve significant improvements in symptom management (38). While, Kristoffersen et al. (41) in their study found no significant improvement in the two groups after the gaming training program was performed. In addition, baseline measurements with the user’s prosthesis were significantly higher than those performed with the machine learning. The study points out that although there was the possibility of using a machine learning-based control in the target population, one of the important limits of the study, as also reported by the authors, was the small number of subjects (41). Finally, Oh et al. (46) found significant differences in caloric intake and Borg rating of perceived exertion (RPE) scale between the SUKIA and NINS groups; in particular, the group that performed SUKIA game training had better caloric consumption and cardiopulmonary endurance. Continuing, Oh et al. (46) found no meaningful differences between the two groups concerning Body Mass Index (BMI) at post-test, suggesting that both types of training intervention were effective in reducing fat mass. Regarding VO2 max, Oh et al. (46) found significant differences between measurements at baseline and post-test. Oh et al.’s (46) analysis showed an improvement in VO2 max in the group that performed SUKIA game training compared to the NINS group but at the same time, both groups improved their parameters compared to baseline, suggesting an important influence on cardiopulmonary function. The SUKIA game training group also showed improvements in the 6MWT compared to the NINS group and, as for VO2 max, both groups showed improvements at post-test compared with baseline. Finally, regarding the post-questionnaire for motivation, perceived exercise effectiveness, and fun, no meaningful differences were observed between the two groups as the subjects who participated in the study were satisfied in both groups (46). The results show the potential of artificial intelligence-enhanced gaming to promote cardiopulmonary health and motivation, though further studies are needed to confirm these effects across diverse populations (46).

#### Bicycle train program, self-monitoring physical activity, and advice based on exercise

Regarding the other three studies, in one a bicycle train program was performed, while in the other two studies self-monitoring physical activity were conducted and advices based on exercise were given (39, 42, 43).

In the study of Mendoza et al. (42) each school intervention had a bicycle train path, which required children 10 to 45 min to complete. They noted that the intervention groups increased the average percentage of daily bicycle trips and in MVPA compared to the control groups. This study emphasizes the excellent ability of artificial intelligence to discriminate one type of activity from another and to analyze specific parameters using accelerometers and GPS (42). Moreover, Bizhanova et al. (39) observed that the significant predictors included (a) sex, (b) rate of weight change by week 4, and (c) rate of adherence to PA goal for week 1. They also observed that higher rates of PA goal adherence for week 1, older age, more years of education, male sex, no history of obstructive sleep apnea (OSA), and being single were related to higher PA goal compliance rates for 52 weeks. Finally, they found that the rate of compliance with the PA goal, at week 1, was similar between the groups, while at week 52, the adherence rate was higher in the group performing self-monitoring of diet, weight, and PA combined with personalized daily feedback messages than in the group performing only self-monitoring of diet, weight, and PA (39). Finally, Nakata et al. (43) developed applications enabling users to record their daily nutrition, mood, exercise, and sleep quality. Over 3 months, Nakata et al. (43) noted a decrease in calorie consumption within the intervention group, declining from 1833 kcal to 1,682 kcal. Nevertheless, although physical activity generally decreased in both groups, the intervention group demonstrated superior preservation, albeit without statistically significant differences (43). This study emphasizes the capability of health applications to monitor user actions and utilize artificial intelligence for focused assessments (43).

## Conclusion

This study evaluates the use, applications, and potential of artificial intelligence across various domains of physical activity and health. The findings suggest that artificial intelligence holds promise in promoting physical activity across diverse populations, including children, adolescents, adults, older adult and individuals with disabilities. Our research found that artificial intelligence, machine learning and deep learning techniques were used: (a) as part of applications to generate automatic messages and be able to communicate with users; (b) as a predictive approach and for gesture and posture recognition; (c) as a control system; (d) as data collector; and (e) as a guided trainer. Future research should prioritize investigating of the incorporation of artificial intelligence, machine learning, and deep learning techniques into physical activity and wellness programs, with a focus on long-term efficacy, accessibility, and scalability.
